# Organic Field-Effect Transistors Based on Chemical-Plated Pt/Ag Electrodes

**DOI:** 10.3390/ma18174130

**Published:** 2025-09-02

**Authors:** Chenyang Zhao, Xiaochen Ren

**Affiliations:** Key Laboratory of Organic Integrated Circuits, Ministry of Education & Tianjin Key Laboratory of Molecular Optoelectronic Sciences, Department of Chemistry, School of Science, Tianjin University Collaborative Innovation Center of Chemical Science and Engineering (Tianjin), Tianjin 300072, China; zhaocy43@tju.edu.cn

**Keywords:** chemical plating, organic field-effect transistor, silver mirror reaction, solution method platinum plating

## Abstract

In this study, we successfully prepared silver electrodes through a silver mirror reaction. By carefully regulating the amount of ammonia complexing agent in the silver–ammonia solution, we effectively suppressed the decomposition of the plating solution while reducing the surface roughness of silver films from 9.22 nm to 4.42 nm. The electrical conductivity of our solution-processed silver layers was nearly one order of magnitude higher than that of conventional inkjet-printed silver electrodes. When applied as source-drain electrodes in organic field-effect transistors (OFETs), these electrodes enabled devices with an average mobility of 0.13 cm^2^/(V·s) and remarkably low mobility variation of only 8.7%. Furthermore, we modified the silver electrodes through chemical platinum plating, achieving a significant 0.74 eV alteration in work function, which demonstrates the great potential of chemical plating for surface functionalization in solution-processed organic electronic devices.

## 1. Introduction

The major advantages of organic electronic materials are that they can dissolve into common organic solvents and that they have excellent optical and electrical properties. The former advantage enables low-cost, large-area device fabrication by using various solution-based processes, including inkjet printing, solution shearing, screen printing, and the roll-to-roll process [1,2,3]. An organic field-effect transistor (OFET) is one of the most important organic electronic devices because it is the building block of organic integrated circuits. OFETs usually consist of a semiconductor layer, a dielectric layer, and electrodes including the source, drain, and gate contact. To realize all types of solution-processed OFET device fabrication, both the semiconductor and dielectric layers are well developed so that they can be prepared using a solution-based method, such as solution shearing [4,5,6,7], screen printing [8,9], inkjet printing [10,11,12,13,14,15], or spin coating [16,17,18]. Solution-processed electrodes mainly rely on printing metal nanoparticle-based ink or conductive polymers such as PEDOT/PSS. The former option always requires a high annealing temperature to vaporize residual solvent and to form a connection between nanoparticles; otherwise, the film’s conductivity is affected. Some metal precursor inks require photochemical reduction to form metal particles, as well as conductive films [19], which increases the complexity of the fabrication process. Surface roughness is another drawback for printed metal nanoparticle electrodes. These electrodes can be annealed at a lower temperature, but the conductivity and stability compared with metal electrodes need to be further improved [20]. To summarize, solution-processed electrodes, in terms of conductivity, surface roughness, and processing temperature, still lag behind metal electrodes prepared via vacuum methods [11,21,22]. Therefore, to realize all solution-processed organic electronic devices while maintaining high performance, developing a solution-based fabrication method to realize high-conductivity, low-processing-temperature electrodes is an urgent challenge, but similar research is rarely reported.

Chemical plating, as a coating method that reduces the content of metal ions in the plating solution to metal and deposits them on the surface of the substrate using a suitable reducing agent in the absence of an applied current, has great potential for preparing metal electrodes via a solution method. The silver mirror reaction, as a chemical silver-plating method, has the advantages of easy access to raw materials, a simple process, and mild reaction conditions compared with other plating solutions, making it very suitable for the low-cost, solution-method preparation of OFET electrodes. However, the silver mirror reaction has always been characterized by the problems of a fast reaction rate, difficulty in controlling the thickness of the silver layer, high surface roughness, and the mismatch of the work function with the energy levels of the p-type organic semiconductor highest occupied molecular orbital (HOMO), which need to be solved [23].

In this study, we report a room-temperature solution-processed silver electrode for OFET applications. We optimize the surface morphology of the chemical-plated silver electrodes via substrate sensitization pretreatment and by adjusting the ratio of the complexing agent, ammonia. The large particles on the surface of the silver layer are reduced, and thus, the roughness is significantly reduced, and the conductivity is close to that of bulk silver. Moreover, the modification of the silver electrode is realized via room-temperature chemical platinum plating, successfully improving the work function of the electrode by 0.7 eV, thus improving the carrier injection of OFET devices. This method is a key step in building sophisticated organic integrated circuits and realizing the full printing of high-performance organic electronics.

## 2. Materials and Methods

### 2.1. Materials

AgNO_3_ standard solution (0.1 mol/L, Jiangtian Chemical Technology Co., Ltd., Tianjin, China), ammonia (2%, Kemio Chemical Reagent Co., Ltd., Tianjin, China), SnCl_2_ (Aladdin Biochemical Technology Co., Ltd., Shanghai, China), concentrated hydrochloric acid (Jiangtian Chemical Technology Co., Ltd., Tianjin, China), glucose (Yuanli Chemical Co., Ltd., Tianjin, China), K_2_PtCl_6_ (Mcklin Biochemical Technology Co., Ltd., Shanghai, China), NaOH (2.5 mol/L, Mcklin Biochemical Technology Co., Ltd., Shanghai, China), ascorbic acid (Yuanli Chemical Co., Ltd., Tianjin, China), DPP-DTT (1-Material Inc., der Stadt Dorval, QC, Canada), and PFBT (Sigma-Aldrich, St. Louis, MO, USA) were used directly without further purification. A heavily n-doped Si wafer with thermally grown 300 nm SiO_2_ was bought from China Electronics Technology Group Corporation.

### 2.2. Sample Preparation

Both the silver mirror reaction and the chemical platinum plating reaction were performed on N-doped silicon wafers with 300 nm SiO_2_, which were previously ultrasonically cleaned with ultrapure water, acetone, and isopropanol, treated with oxygen plasma for 10 min, and sensitized in a hydrochloric acid solution (0.58 × 10^−3^ mol/L) of SnCl_2_ for 1 min. The silver mirror reaction was carried out in a silver–ammonia solution and a glucose solution. The silver–ammonia solution was prepared by proportionally mixing AgNO_3_ standard solution with ammonia (2%), and the metal layer was patterned by photolithography. The chemical platinum plating reaction was carried out based on the silver mirror reaction. After a short 40–50 s period of the silver mirror reaction and photolithographic patterning, the samples were immersed in a reaction solution of K_2_PtCl_6_ and NaOH mixed with ascorbic acid, and heated to 50 °C for the reaction. OFET devices were prepared by spin-coating DPP-DTT (5 mg/mL, in CHCl_3_) on the samples of two different electrodes and annealing at 200 °C for 5 min.

### 2.3. Characterization

The surface morphology, thickness, and surface potential of the metal layers were tested by atomic force microscopy (Bruker, Dimension Icon, Park Systems Corporate, Suwon, Republic of Korea), the sample elemental analysis was performed by scanning electron microscopy (SU 8010, Hitachi America, Ltd., Santa Clara, CA, USA), and the electrical properties of the OFET devices were tested by a digital source meter (Keithley 2636B, Tektronix, Inc., Beaverton, OR, USA).

## 3. Results

### 3.1. Optimization of the Silver Mirror Reaction Surface Morphology

We first optimized the processing conditions of the silver mirror reaction to improve the surface quality, thus satisfying the requirements of contact electrodes for OFET devices.

**Figure 1:**
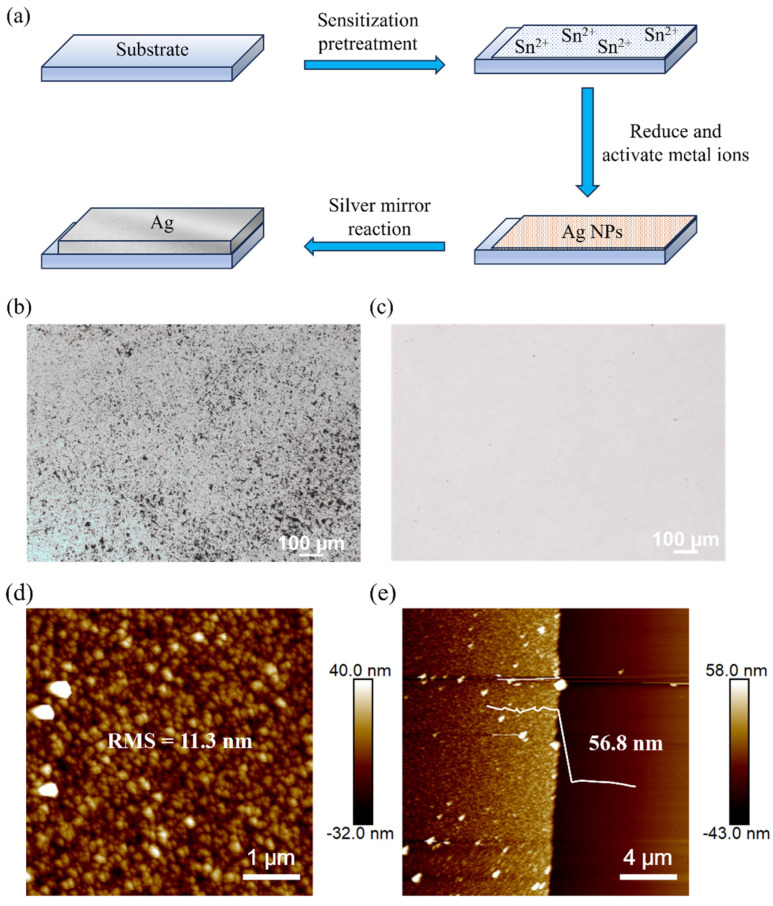
(**a**) Schematic diagram of the process of preparing a silver layer through the silver mirror reaction. (**b**) Optical image of the silver layer obtained from the reaction without sensitization treatment of the substrate. (**c**) Optical image of the silver layer obtained from the reaction after sensitization treatment of the substrate. (**d**) Roughness. (**e**) Thickness.

The silver mirror reaction generally involves immersing the cleaned substrate in the solution for a certain period of time to obtain a well-deposited Ag layer, but the metal layer tends to be porous-like, affecting the electrical conductivity and surface roughness of Ag. However, after pretreating the substrate surface with the hydrochloric acid solution of SnCl_2_ and reducing the layer of Ag nanoparticles (NPs) on the surface as a reaction site for the silver mirror reaction, Ag precipitated via the silver mirror reaction can be better deposited on the surface of the substrate. Then, a densely packed Ag metal layer is obtained after the silver mirror reaction (the preparation process is shown in Figure 1a). Figure 1b,c are the optical images of the silver film formed without and with sensitization, respectively, and it is obvious that most of the holes or large particles in the Ag layer are eliminated after sensitizing the substrate for the silver mirror reaction, indicating that the sensitization treatment greatly improves the film quality of the silver mirror reaction.

Typically, the silver mirror reaction is conducted quickly at room temperature, and the solution rapidly precipitates silver within 1 min. It then greatly increases the surface roughness of the resulting layer. To avoid the rapid decomposition of the plating solution, we increased the amount of ammonia complexing agent involved in preparing the silver–ammonia solution, thus increasing the stability of the plating solution, so that Ag is only precipitated and deposited on the surface of the substrate during the reaction period.

When preparing silver–ammonia solutions, initially adding ammonia to the AgNO_3_ solution produces a brownish Ag_2_O precipitate, and continuing to add ammonia dissolves the precipitate and results in Ag(NH_3_)_2_OH, the silver–ammonia solution. According to the concentration of the AgNO_3_ solution and ammonia used in our experiment, the precipitate is just dissolved when the volume ratio of the AgNO_3_ solution to ammonia is 5:1. The silver mirror reaction was carried out with the silver–ammonia solution prepared at a ratio of 5:1 for 5 min. The thickness of the Ag layer and the surface morphology were characterized by the AFM, as shown in Figure 1d and Figure 1e, respectively. The thickness was found to be 56.8 nm, and the surface roughness was 11.3 nm. Although the surface roughness of the prepared Ag film was already lower than the roughness of the Ag layer obtained via the silver mirror reaction in the previous report [23], it was still too high when using the electrodes in OFET devices. In addition, the presence of large particles on the surface, as evident from the AFM images, needed further optimization.

**Figure 2:**
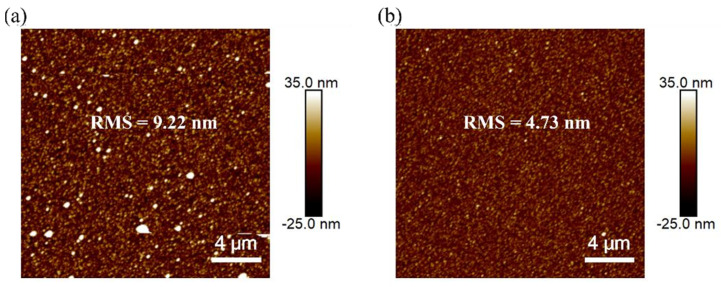
AFM image of Ag when the ratio of silver nitrate to ammonia is (**a**) 5:1 and (**b**) 5:4.

We tried to increase the amount of ammonia proportionally to improve the surface morphology of the Ag layer, aiming to reduce the presence of large particles and to decrease the surface roughness. The volume ratio of the AgNO_3_ solution to ammonia was adjusted to 5:2, 5:3, 5:4, 5:5, and 5:6, and the silver mirror reaction was carried out for 5 min, with the other experimental conditions kept unchanged. The corresponding surface roughness and thickness values are listed in Table 1. As shown in the table, when the V_silver nitrate_/V_ammonia_ ratio increased to 5:4, the roughness and thickness of the Ag layer reached their minimum values at about 4.5 nm and 39 nm, respectively, and no longer changed. Next, for a ratio of 5:1, we intentionally reduced the reaction time to 3 min to keep the film thickness at around 39 nm, as shown in the bottom row of Table 1, allowing for a direct comparison of the surface roughness at the same film thickness across different ammonia ratios. The corresponding AFM surface scanning images of these two samples are shown in Figure 2. Compared with the data at a ratio of 5:1, the roughness was reduced significantly, and large particles were also eliminated, indicating that the excess amount of ammonia was key to stabilizing the plating solution and, thus, to reducing the surface roughness. The optimized Ag film produced via the silver mirror reaction was ready for the subsequent fabrication of OFET devices.

### 3.2. Conductivity of the Silver Electrode

**Figure 3:**
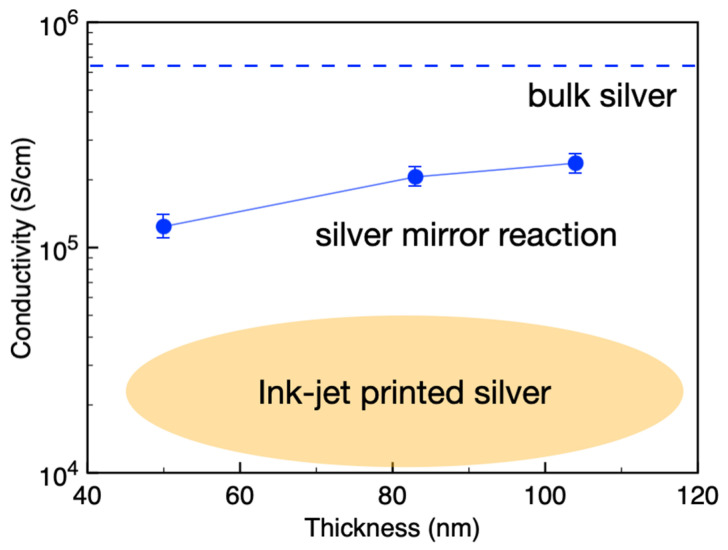
Conductivity of Ag films corresponding to different thicknesses.

Samples with Ag film thicknesses of 50.7 nm, 83 nm, and 104 nm were prepared by varying the silver mirror reaction time for resistivity and conductivity measurements, and the results are shown in Figure 3. The conductivity monotonically increases from 1.24 × 10^5^ S/cm to 2.37 × 10^5^ S/cm with increasing film thickness, increasing in the same order of conductivity as bulk silver (6.25 × 10^5^ S/cm, dashed line in Figure 3).

Compared with inkjet-printed silver electrodes, the conductivity of inkjet-printed electrodes after multilayer printing and high-temperature sintering is usually lower than 5 × 10^4^ S/cm [24], which is nearly an order of magnitude smaller than that of silver electrodes obtained via the silver mirror reaction. This proves that the silver mirror reaction is a promising candidate for generating high-quality solution-processed electrodes with conductivity close to that of bulk silver through processing at room temperature.

In addition, we performed a standard tape test on the Ag film prepared via the silver mirror reaction and vacuum evaporation on glass and silicon substrates, respectively. Before tape attachment, an English letter was written on the film using a marker pen. Consequently, Kapton tape was attached to the film and then peeled off gradually from one side. The test results are shown in Figure S1 in the Supplementary Materials. All of the evaporated Ag film was detached using Kapton tape on various substrates, while the chemical-plated Ag was not affected. After comparison with the vacuum-evaporated samples, we proved that the coating obtained via the silver mirror reaction had better adhesion than the thermally evaporated Ag film.

### 3.3. OFET Performance Characterization

**Figure 4:**
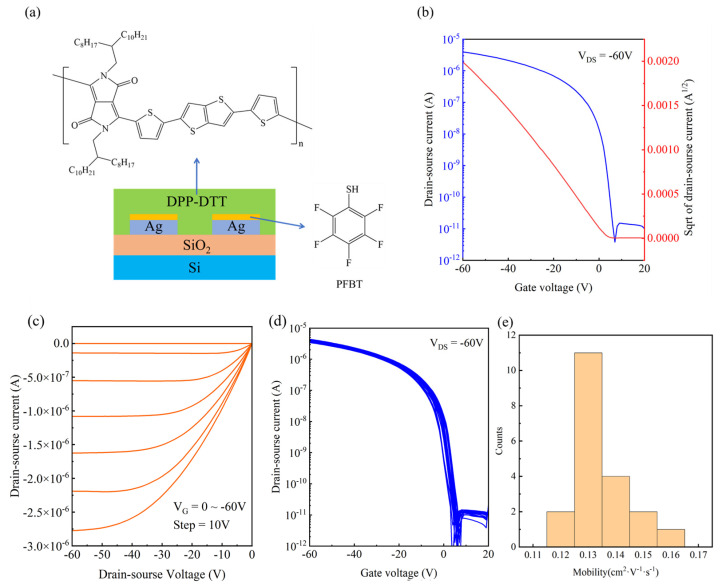
(**a**) Schematic diagram of the OFET device structure based on the electroless silver-plated electrode. (**b**) Transfer characteristic curve. (**c**) Output curve. (**d**) Transfer characteristic curves of 20 OFET devices. (**e**) Distribution diagram of the saturation mobility of 20 OFET devices.

To better evaluate the performance of silver mirror reaction Ag electrodes, bottom-gate bottom-contact OFET devices were constructed via photolithography, patterning Ag as the source-drain electrodes on a SiO_2_/Si substrate and using DPP-DTT as the semiconductor. Since the work function of Ag is 4.26 eV, which does not match the HOMO energy level (−5.2 eV) of the semiconductor, the silver surface was modified with a pentafluorobenzenethiol (PFBT) self-assembly monolayer to increase its work function [25] for better carrier injection of OFETs. PFBT could form an Ag-S bond with Ag, thereby enhancing the work function of the electrodes [26]. The device structure is schematically shown in Figure 4a. The device performance was evaluated by measuring transfer I-V curves and output I-V curves, as shown in Figure 4b,c.

The transfer I-V curve exhibits typical p-type transistor characteristics with an on/off current ratio of 5 × 10^5^, the threshold voltage of 4.47 V is obtained via linear regression analysis, and the saturation mobility of the device is 0.16 cm^2^/(V·s). This is on the same order of magnitude as the mobility of the solution-method electrodes reported in related reports using a polymer as the semiconductor [27]. In the output curve in Figure 4c, the device shows good linear behavior in the low-source-drain voltage region, indicating that the device carriers are well injected after modification using PFBT. Subsequently, we performed electrical measurements on 20 devices to evaluate the device uniformity. Figure 4d shows the transfer I–V curves of all 20 devices, suggesting excellent device uniformity. The mobility values of the 20 devices were calculated, and their distribution is shown in Figure 4e. An average mobility of 0.13 cm^2^/(V·s) and a coefficient of variation (CV) of 8.7% were obtained, indicating that the OFET devices based on chemical-plated silver electrodes exhibit very good device uniformity.

### 3.4. Chemical-Plated Platinum Electrode

**Figure 5:**
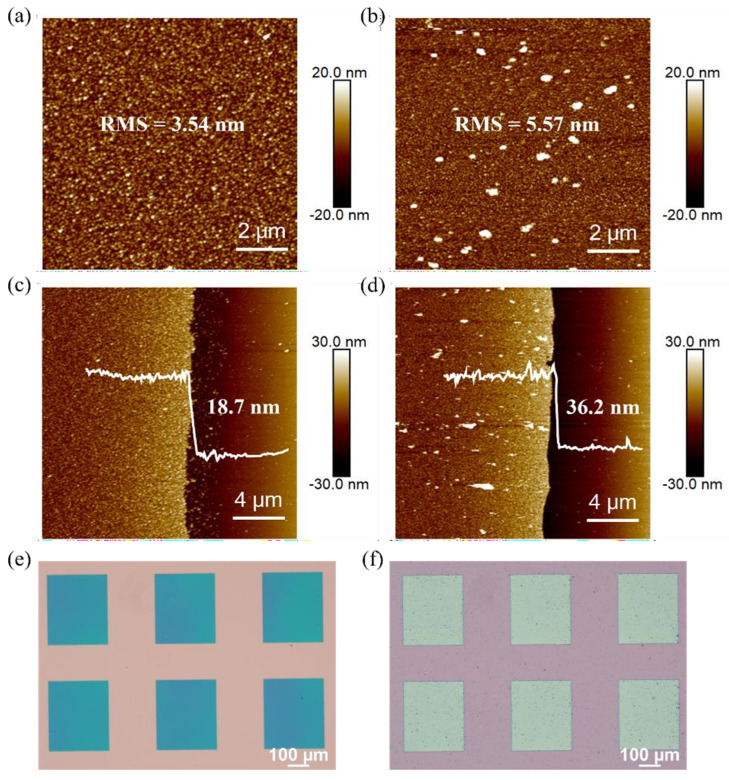
(**a**) Surface roughness and (**c**) thickness of the electroless silver plating layer in a short time. (**b**) Surface roughness and (**d**) thickness after electroless platinum plating. Optical images (**e**) before and (**f**) after electroless platinum plating.

To demonstrate the functionalization ability of the chemical plating method, we further performed chemical platinum plating based on a chemical-plated Ag film to alter the work function of silver electrodes. The chemical-plated Ag film served as the initial catalyst, as well as the deposition pattern for chemical platinization. As the catalyst, a thinner film thickness and a porous-like surface morphology were preferred. Therefore, the silver–ammonia solution with a ratio of AgNO_3_ solution to ammonia of 5:1 was chosen as the reaction solution for short-time chemical silver plating for 40~50 s, and an Ag layer with a thickness of 15~20 nm was obtained, which could act as a catalyst for chemical platinizing and did not fall off during the patterning process. The surface morphology and thickness of the Ag layer are shown in Figure 5a and Figure 5c, respectively, where the thickness is only 18.7 nm due to the short reaction time, and the surface roughness is 3.54 nm.

For the subsequent chemical platinization reaction, we used K_2_PtCl_6_ as the main salt, NaOH as the complexing agent to form Na_2_Pt(OH)_6_ solution, and ascorbic acid as the reducing agent. These two agents were mixed and preheated at 50 °C for 30 min to initiate the redox reaction, and then the Ag film sample was put into the reaction solution to begin platinization. The surface morphology and thickness characterization of the final platinized layer are shown in Figure 5b,d. The thickness increased from 18.7 nm to 36.2 nm, and the surface roughness also increased from 3.54 nm to 5.57 nm. Since the chemical platinizing solution was generally not as stable as the chemical silver-plating solution, platinum precipitated from the plating solution during the reaction and contaminated the metal surface, resulting in large particles becoming attached and increasing the surface roughness (Figure 5d).

Because chemical platinum plating occurs only where there is silver present, the pattern of the Pt-covered electrode is directly defined by the pattern of the silver film before platinization. The optical images of the silver electrodes before and after chemical platinum plating are shown in Figure 5e and Figure 5f, respectively.

**Figure 6:**
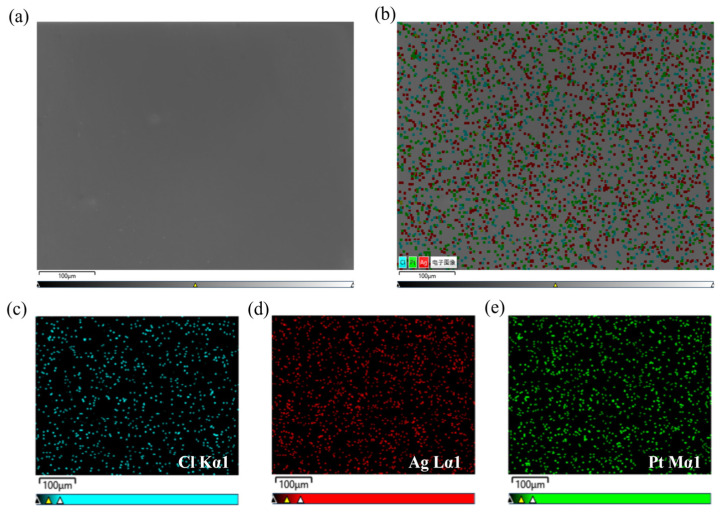
(**a**) SEM image of the platinum-plated sample. (**b**) EDS image, the meaning of “电子图像” in the picture is electronic image. Corresponding elemental distributions: (**c**) Cl, (**d**) Ag, and (**e**) Pt.

To verify the successful deposition of Pt on the silver surface, EDS energy spectroscopy was used to analyze and characterize the elemental species of the sample surface. Surface scanning of the sample was performed to obtain the distribution of each element, as shown in Figure 6. The scale bar in Figure 6a,b is 100 μm. The elemental distributions of Cl, Ag, and Pt in the sample after chemical platinum plating correspond to the blue, red, and green colors, respectively. The elemental contents of Ag and Pt are almost the same, with mass fractions of 48.73% and 50.56% weight ratio, respectively, consistent with the changes in the thickness of the plated layer mentioned above. The elemental content of chlorine is only 0.71%; this small amount of chlorine may be related to the residual chlorine ions in the composition of the plating solution. In Figure 6d,e, the distribution of silver and platinum is very uniform, proving the successful growth of metallic platinum. The success of the chemical-plated platinum method proves the feasibility of preparing platinum electrodes via a solution method, avoiding the use of high-energy-consumption and harsh conditions of the electron beam evaporation (EBE) method [28].

### 3.5. Variation in Electrode Work Function

**Figure 7:**
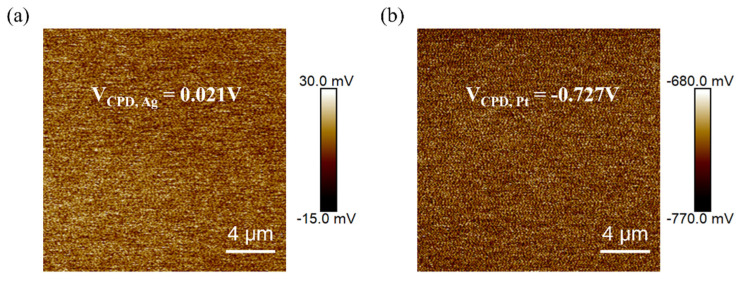
(**a**) KPFM image of the coating layer obtained by the silver mirror reaction; (**b**) KPFM image of the coating layer after the electroless platinum plating reaction.

We performed KPFM characterization of the sample plating after 40 s of the silver mirror reaction and 3 min of the chemical platinum plating reaction, as shown in Figure 7. Before proceeding with characterizing the samples, the work function of the platinum–iridium probe was calibrated using a standard sample of highly oriented pyrolytic graphite (HOPG) with a work function of 4.6 eV, yielding *W*probe = 4.76 eV.

As can be seen in Figure 7, the surface potential differences between the silver-plated and platinum-plated samples are 0.021 V and −0.727 V, respectively, and the respective measured work functions are 4.74 eV and 5.48 eV, according to the formula *W*sample = *W*probe *− eV*_CPD_. The value of 5.48 eV is very close to that of platinum (5.65 eV), which may be lower than the normal range under the influence of the test environment. This work function verifies the successful deposition of Pt. The work function of the silver-plated film is successfully altered by 0.74 eV via platinum plating, demonstrating the great potential of surface functionalization through the chemical plating method.

As shown in Figure 7, the surface potential differences for the silver-plated and platinum-plated samples are 0.021 V and −0.727 V, respectively, and the respective measured work functions are 4.74 eV and 5.48 eV according to the formula *W*sample = *W*probe *− eV*_CPD_. The value of 5.48 eV is very close to that of platinum (5.65 eV), which may be lower than the normal range under the influence of the test environment. This work function verifies the successful deposition of Pt. The work function of the silver-plated film is successfully altered by 0.74 eV via platinum plating, demonstrating the great potential of surface functionalization through the chemical plating method.

### 3.6. Chemical-Plated Platinum Electrode OFET Device

**Figure 8:**
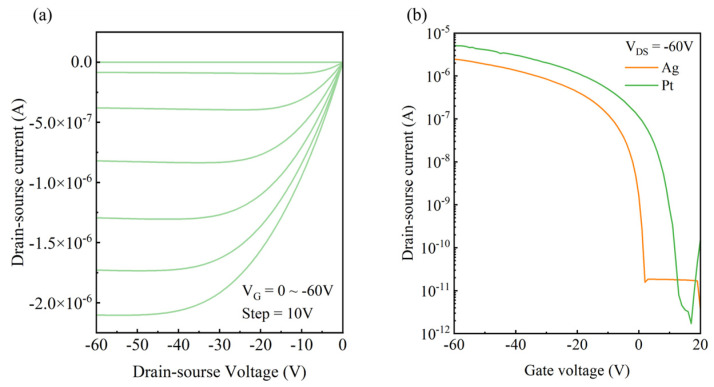
(**a**) Output curve of the platinum-plated electrode. (**b**) Comparison of the transfer characteristic curves between the platinum-plated electrodes and the pure silver electrodes.

OFETs based on Pt/Ag electrodes were fabricated to improve charge injection compared to pure Ag electrodes. SiO_2_/Si was used as the substrate, on which the silver mirror reaction, photolithography patterning, and chemical platinum plating were carried out in sequence to complete source-drain electrode fabrication. DPP-DTT was used as the organic semiconductor layer through spin coating to construct the bottom-gate bottom-contact OFET device. The output and transfer I–V curves are shown in Figure 8a and Figure 8b, respectively. The output I–V curve shows negligible nonlinear phenomena in the small-drain-source voltage region, suggesting that there is a very small injection barrier at the electrode/semiconductor contacts. The green curve in Figure 8b represents the device performance with Pt/Ag electrodes. The saturation mobility of the device was found to be 0.16 cm^2^/(V·s), which is very similar to the average mobility of the PFBT-modified silver electrode device. The on/off current ratio was about 10^6^, and the threshold voltage was 9.58 V, as obtained via linear regression analysis. To verify the effect of the Pt-modified electrode, a control device with pure Ag electrodes without a PFBT self-assembly monolayer was measured, as shown by the orange curve in Figure 8b. The saturation mobility of the pure silver electrode device is 0.09 cm^2^/(V·s), and the threshold voltage is 2.43 V. In comparison, the saturation mobility of the platinum-plated electrode OFET device is improved by more than 70%. This was mainly attributed to the high work function of Pt that reduced the charge injection energy barrier. The variation in the threshold voltage may be related to the change in the dielectric layer interface trap state density after the chemical platinum plating reaction [29,30]. The contact resistance results further prove the improvement in charge injection. We used the TLM method to measure the contact resistance of the three types of electrodes, as shown in Figure S2. The final contact resistances (R_c_·W) corresponding to Ag, Ag-PFBT, and Ag-Pt were 27.8, 2.17, and 1.92 kΩ·cm, respectively. This suggests that PFBT plays a critical role in improving charge injection. The contact resistance was largely reduced after the growth of the PFBT self-assembly monolayer. The chemical Pt coating also significantly improved the charge injection compared to bare Ag electrodes.

In addition, the mobility of OFETs based on pure silver electrodes was nearly one order of magnitude higher than that of inkjet-printed silver electrodes [24]. The above results indicate that compared with mature-printed electrodes, the preparation process for chemical-plated electrodes is simpler, and the device performance is comparable or slightly improved.

## 4. Conclusions

In this work, we developed high-performance solution-processed electrodes through an optimized silver mirror reaction and chemical platinum plating. Substrate sensitization and ammonia ratio optimization reduced surface roughness to 4.42 nm while achieving conductivity approaching bulk silver. The electrodes enabled OFETs with 0.13 cm^2^/(V·s) mobility and excellent uniformity (8.7% variation). Chemical platinum plating successfully increased the work function by 0.74 eV, yielding devices with 0.16 cm^2^/(V·s) mobility. This approach provides a low-temperature, solution-based alternative to vacuum deposition methods, offering great potential for large-area fabrication of high-performance organic electronic devices.

## Figures and Tables

**Table 1:** The roughness and thickness of the silver layers obtained with different amounts of ammonia.

V_silver nitrate_:V_ammonia_	Reaction Time/min	Roughness/nm	Thickness/nm
5:1	5	11.3 ± 2.56	56.8 ± 4.7
5:2	5	6.58 ± 1.79	45.7 ± 3.9
5:3	5	6.01 ± 1.26	43.8 ± 2.2
5:4	5	4.73 ± 1.14	39.9 ± 2.3
5:5	5	4.42 ± 0.64	38.7 ± 1.9
5:6	5	5.07 ± 0.81	39.6 ± 2.1
5:1	3	9.22 ± 1.24	38.8 ± 3.6

## Data Availability

The original contributions presented in the study are included in the article/Supplementary Material. Further inquiries can be directed to the corresponding author.

## Supplementary Materials

The following supporting information can be downloaded at: https://www.mdpi.com/article/10.3390/ma18174130/s1, Figure S1. Standard tape tests of chemical plated Ag and thermally evaporated Ag films; Figure S2. Contact resistance of different electrodes: (a) Ag; (b) Ag-PFBT; (c) Ag-Pt.
