# Supplementary material for: Organic Field-Effect Transistors Based on Chemical-Plated Pt/Ag Electrodes
Source: Materials (Basel). 2025 Sep 2;18(17):4130. doi: 10.3390/ma18174130 (PMC12429967; doi:10.3390/ma18174130)
Supplement: Supplementary file 1 [file materials-18-04130-s001.zip › materials-3784105-supplementary.pdf]

Supplementary Information

Organic Field-Effect Transistors Based on Chemical-Plated Pt/Ag Electrodes

Chenyang Zhao and Xiaochen Ren *

Key Laboratory of Organic Integrated Circuits, Ministry of Education & Tianjin Key Laboratory of Molecular Optoelectronic Sciences, Department of Chemistry, School of Science, Tianjin University Collaborative Innovation Center of Chemical Science and Engineering (Tianjin), Tianjin 300072, China; zhaocy43@tju.edu.cn

* Correspondence: renxiaochen@tju.edu.cn

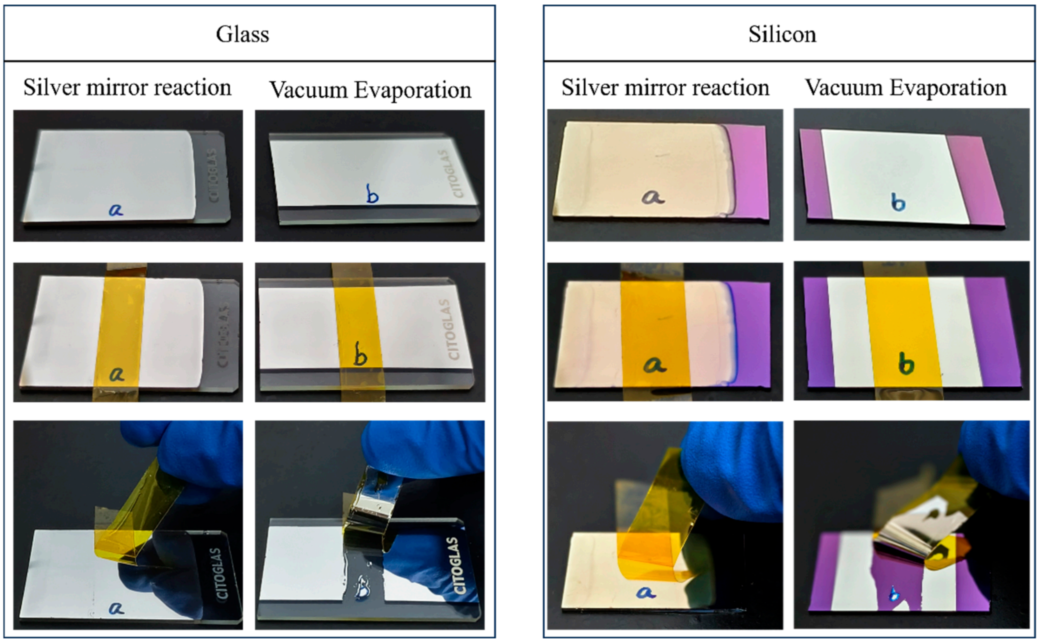

Figure S1. Standard tape tests of chemical plated Ag and thermally evaporated Ag films.

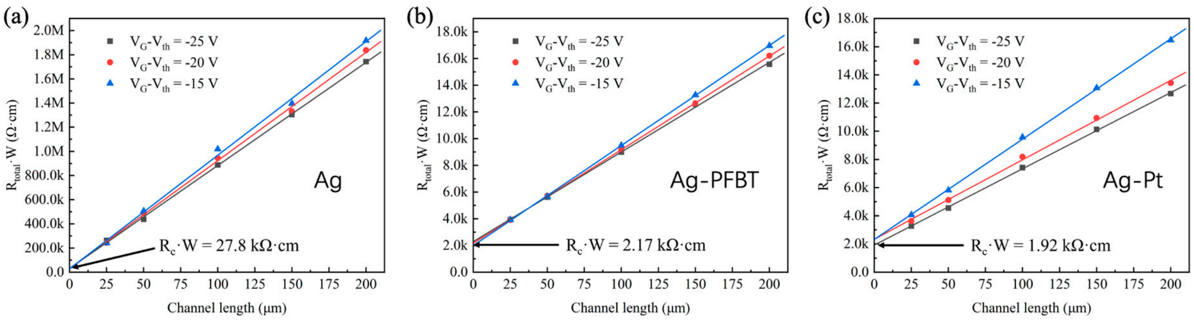

Figure S2. Contact resistance of different electrodes: (a) Ag; (b) Ag-PFBT; (c) Ag-Pt.
